# Exploring the Imbalance of Periodontitis Immune System From the Cellular to Molecular Level

**DOI:** 10.3389/fgene.2021.653209

**Published:** 2021-03-26

**Authors:** Longfei He, Lijuan Liu, Ti Li, Deshu Zhuang, Jiayin Dai, Bo Wang, Liangjia Bi

**Affiliations:** ^1^Department of Stomatology, The Fourth Affiliated Hospital of Harbin Medical University, Harbin, China; ^2^Department of Stomatology, Weifang People’s Hospital, Weifang, China; ^3^Department of Oral Biological and Medical Sciences, Faculty of Dentistry, University of British Columbia, Vancouver, BC, Canada

**Keywords:** periodontitis, DEGs, crosstalk gene, PPI, immune system

## Abstract

Periodontitis is a common chronic inflammatory disease of periodontal tissue, mostly concentrated in people over 30 years old. Statistics show that compared with foreign countries, the prevalence of periodontitis in China is as high as 40%, and the prevalence of periodontal disease is more than 90%, which must arouse our great attention. Diagnosis and treatment of periodontitis currently rely mainly on clinical criteria, and the exploration of the etiologic criteria is relatively lacking. We, therefore, have explored the pathogenesis of periodontitis from the perspective of immune imbalance. By predicting the fraction of 22 immune cells in periodontitis tissues and comparing them with normal tissues, we found that multiple immune cell infiltration in periodontitis tissues was inhibited and this feature can clearly distinguish periodontitis from normal tissues. Further, protein interaction network (PPI) and transcription regulation network have been constructed based on differentially expressed genes (DEGs) to explore the interaction function modules and regulation pathways. Three functional modules have been revealed and top TFs such as EGR1 and ETS1 have been shown to regulate the expression of periodontitis-related immune genes that play an important role in the formation of the immunosuppressive microenvironment. The classifier was also used to verify the reliability of periodontitis features obtained at the cellular and molecular levels. In conclusion, we have revealed the immune microenvironment and molecular characteristics of periodontitis, which will help to better understand the mechanism of periodontitis and its application in clinical diagnosis and treatment.

## Introduction

Periodontitis is a chronic inflammatory disease with complex pathogenesis. It will gradually cause the loss of periodontal ligament and alveolar bone, and eventually cause tooth loss (Hajishengallis, 2015; Hajishengallis and Korostoff, 2017). As one of the most prevalent chronic inflammatory diseases in the world, periodontitis directly affects more than 11% of the global population. According to the National Health and Nutrition Examination Survey of the United States, nearly half of American adults suffer from periodontitis, which is a huge number and seriously affects the quality of life of individuals (Eke et al., 2015). Recent studies have shown that periodontitis not only affects the periodontal area, it is also the cause of other systemic diseases, such as rheumatoid arthritis, atherosclerosis and cerebrovascular diseases (Genco and Van Dyke, 2010; Kebschull et al., 2010; Lundberg et al., 2010). In addition, studies have found that as many as one-third of the periodontitis mutations in the population are caused by genetic factors, and the more severe the periodontitis, the stronger the heritability (Nibali et al., 2019).

Studies have confirmed that infection of external microbial flora is an important factor in causing periodontitis. Earlier, Porphyromonas gingivalis was considered to be the cause of periodontitis. But with the advancement of science, we have found that periodontitis induced by Porphyromonas gingivalis requires the presence of symbiotic flora (Hajishengallis et al., 2011). Although with the study of the etiology of periodontitis is more detailed, the most important is the local microbiota and host immune response (Hajishengallis, 2014a). Under normal physiological conditions, the host periodontal local immune response and microbes are in a delicate balance state, realizing routine monitoring of the flora (Graves et al., 2019). However, once the pathogen colonizes the periodontal area, it will significantly increase the number and destructiveness of the microbial flora, breaking the original dynamic balance (Hajishengallis et al., 2012). Under this condition, the immunity will be over-activated and immune invasion will occur, thereby destroying the activity of periodontal tissues. Different from the immune evasion of other pathogens (Cyktor and Turner, 2011), the periodontitis flora interacts with the immune response to improve its adaptability and use the tissues destroyed by inflammation to obtain nutrients (Hajishengallis,2014a,b).

After all, the process of periodontitis is caused by the dynamic imbalance of local immunity and microbial community. Immune invasion will cause the activation of osteoclasts, which will resorb alveolar bone (Belibasakis and Bostanci, 2012). The abnormality of cytokines in the host immune response has been revealed in previous studies (Pan et al., 2019). Cytokines are key regulators of local tissue homeostasis and inflammatory processes, playing a role in the first wave of the host’s response to pathogens and stimuli, and connect tissue cells with lymphocytes and helper cell populations to work together (Graves, 2008). The immune imbalance of periodontitis leads to systemic inflammation (Hajishengallis, 2015), and a large number of studies on the pathogenesis of periodontitis involve changes in host immunity. But so far, no scholar has fully revealed the immune imbalance of periodontitis from cells to molecules. In this study, we will reveal the new pathogenesis of periodontitis and the abnormal molecular mechanism through protein interaction analysis and targeted regulation analysis of related immune genes.

## Materials and Methods

### Data Collection

The expression profile and sample annotation of periodontitis diseases was downloaded from the GEO database^1^, including three series GSE10334 (183 periodontitis and 64 normal), GSE16134 (241 periodontitis and 69 normal) and GSE23586 (3 periodontitis and 3 normal, Table 1). Next, we download all immunosuppressive-related genes from DisGeNET (Pinero et al., 2017)^2^ and HisgAtlas (Liu et al., 2017)^3^. In addition, we searched for drugs related to immunosuppressive agents from Drugbank (Wishart et al., 2018)^4^ obtained 311 immunosuppressive-related drugs, and then downloaded immunosuppressive-related genes. We merged the immunosuppressant-related genes obtained from the above three databases, and obtained a total of 1,332 genes. We started from BIND (Gilbert, 2005), BioGRID (Oughtred et al., 2019)^5^, MINT (Chatr-aryamontri et al., 2007)^6^, HPRD (Goel et al., 2012)^7^, IntAct (Kerrien et al., 2012)^8^, and OPHID (Brown and Jurisica, 2005)^9^ database to download protein interaction data, and integrate these data. We also downloaded immune-related genes from the InnateDB (Breuer et al., 2013)^10^ database. The transcription factor (TF) and target gene relationship from the relevant transcription regulation databases TRRUST v2 (Yang et al., 2018)^11^ and ORTI (Vafaee et al., 2016)^12^.

**TABLE 1:** Description of microarray profiles in gingival tissue.

GEO series	Periodontitis Sample	Normal sample	Tissues	Platforms	Citation (PMID)
GSE10334	183	64	Gingival	Affymetrix; GPL570	18980520
GSE16134	241	69	Gingival	Affymetrix; GPL570	19835625, 24646639
GSE23586	6	6	Gingival	Affymetrix; GPL570	21382035

### Immune Cell Distribution Analysis

We have preprocessed the expression matrices of the three series of GSE10334, GSE16134, and GSE23586 and extracted the expression profiles of immunosuppressant-related genes in periodontitis diseases for immune invasion analysis. CIBERSORT (Newman et al., 2015) could be used to predict the infiltrating immune cells that are highly related to periodontitis disease. Here, we used the R version of CIBERSORT instead of the web version, taking into account the user-friendly operation. CIBERSORT has four parameters including the reference set that can be downloaded at https://cibersort.stanford.edu/download.php, the expression matrix we prepared, perm that is the number of permutations when calculating the *p*-value and is set to 1,000, and QN that is whether to perform quantile normalization and is set to TRUE taking into account the microarray expression data. In order to see more group differences in the fraction of cell types other than plasma cells, we further transformed the raw cell fractions into the log ratio of log (plasma_cell_fraction + 1e-3)/log (cell_fraction + 1e-3). We also combined previous studies on periodontitis clustering to explore the differences in the immune microenvironment between periodontitis subtypes.

### Differential Expression Analysis and Functional Enrichment Analysis

We consider the sample size of each series in the downloaded data, so we only perform differential expression analysis on the downloaded sample data of GSE10334 and GSE16134. In data preprocessing, missing values of the expression matrix were filled by zero value. Further, the gene expression values were log2-transformed to be suitable for differential expression analysis. The limma package was used to measure gene expression variation between periodontitis and normal samples. We defined the cutoff of gene *p*-value as 0.05 and the cutoff of fold-change as 1.5 (Demmer et al., 2008), which filtered out differentially expressed genes (DEGs). The clinical variables were not include in the DEG identification pipeline Next, we integrate the significant DEGs of these two series of samples into a multi-gene set list, and use the compareCluster_go() function of the latest clusterProfiler package of the R language to perform GO function and KEGG enrichment on the data set, and set threshold *p* < 0.05.

### Construction of PPI Network and Transcriptional Regulatory Network

We extract gene pairs that interact with DEGs from the PPI data, and use the network rendering tool Cytoscape to map the differential gene PPI data. Further, the MCODE module of Cytoscape were used to screen the significant function modules in the DEG PPI network (parameter selection: Degree cutoff: 5, Node score cutoff: 0.2, K-core: 2, and Max. depth: 100), and used the network analysis tool to analyze the topological properties of the network (Degree, Average Shortest Path Length, Betweenness Centrality, Closeness Centrality, Clustering Coefficient, Topological Coefficient). We use differentially expressed immune genes as crosstalk genes, and extract the PPI relationship pairs of these crosstalk genes, and use Cytoscape to construct the crosstalk gene PPI network. We defined the modules identified in the PPI network of immune-related genes that were masked in the PPI network constructed directly using DEGs as New-module of immune function. We extracted the TF-target relationship pairs related to the crosstalk gene and constructed the TF-target network using Cytoscape software. We then analyzed the topological properties of the network, and extracted the top 10 genes of outdegree and indegree, respectively, as key periodontitis related genes.

### Build the Classifier

We constructed periodontitis disease classifiers with significantly different infiltration of immune cells as the characteristic and New-module functional gene in the crosstalk gene PPI network as the characteristic. The former uses the fraction of immune cell identified by CIBERSORT and the latter uses gene expression data. Here, we consider two classification algorithms, including decision tree and SVM, to build the model. We randomly select 70% of the samples in GSE10334 as the training set, and the remaining 30% as the test set, and use the data of the GSE16134 and GSE23586 series as the validation sets. Further, we combine the possibility provided by the classifier and the true sample label to measure the performance of the classifier. In order to understand the generalization ability of the model, we introduced fivefold cross-validation. We use the pROC package and plot function of the R language to display the ROC curve to evaluate the effectiveness of the model.

## Results

### Immune System Imbalance at the Cellular Level

#### Immune Cell Infiltration in Periodontitis

**FIGURE 1:**
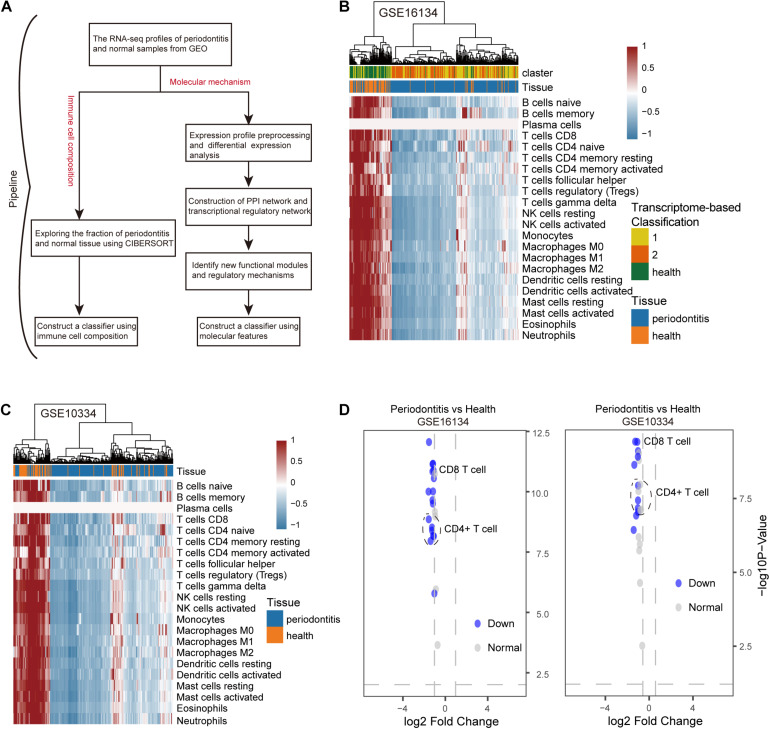
The distribution of 22 types of immune cells in periodontitis and healthy samples. **(A)** Diagram of the multiple components and workflows of pipeline. **(B)** The heatmap represents the fraction of immune cells for the GSE16134 series. The horizontal axis is the immune cell type and the vertical axis is the sample. **(C)** The same as in **(B)** but for GSE10334. **(D)** The volcano plot represents the immune cells with significantly different gene expression levels between periodontitis and healthy samples for the GSE10334 and GSE16134 series.

We developed a computational pipeline to analyze the gene expression profile of periodontitis disease (Figure 1A). In this study, we selected microarray profiles of the GSE10334 and GSE16134 series with sufficient periodontitis and normal samples for immuno-infiltration analysis of gingival tissue. After quality control and normalization, we obtained two processed expression profiles. Here, we used the CIBERSORT method to predict the infiltration of immune cells in periodontitis disease. We obtained the fraction of 22 immune cell types in these samples. We further transformed the raw cell fractions in order to see more group differences in the fraction of cell types (Figures 1B,C and Supplementary Table S1). We found decreased levels of immune infiltration during the malignant transformation of normal tissue to periodontitis that was verified in both series of samples, which indicates that periodontitis tissue undergoes immunosuppressive microenvironment. By combining this with previous studies (Kebschull et al., 2014), we found that the level of immune infiltration in type 1 periodontitis was superior to that in type 2 periodontitis (Figure 1B), indicating that type 1 periodontitis may be more suitable for immune targeted therapy. We found that the fraction of CD4+/CD8+ T cells in periodontitis tissue was significantly depressed (Figure 1D), which might be one of the factors contributing to the suppression of the immune microenvironment in periodontitis tissues.

#### Construct a Classifier Based on Immune Cells

**FIGURE 2:**
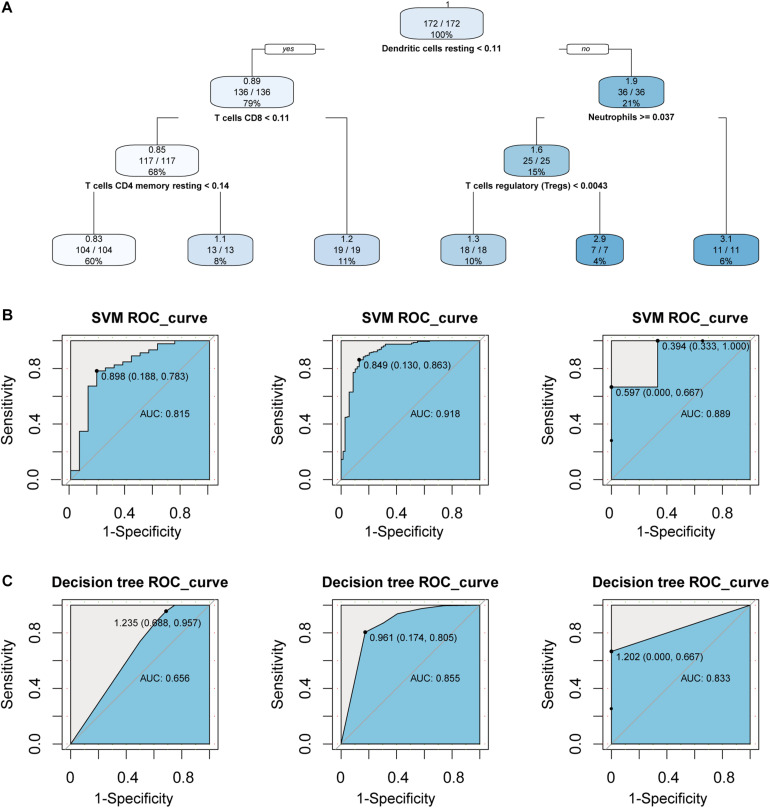
Construct a classifier with significantly different distribution of immune cells. **(A)** This picture is the decision tree diagram of the decision tree classifier. **(B)** The ROC curve represents the area under curve (AUC) of the test set and validation set for SVM classifiers. **(C)** The same as in **(B)** but the Decision tree.

In order to consider whether immune cells with significant changes in fraction can represent the overall difference between periodontitis and normal patients, we constructed a classifier based on the significantly different distribution of immune cells. The two machine learning methods, including Decision tree and SVM, were used to build the classifier model, and the training set, test set, and validation set were also scientifically allocated. In the model constructed by the decision tree, dendritic cell, neutrophils, and CD4+/CD8+T cell were used as important screening indicators to control sample filtering (Figure 2A). In order to predict the accuracy of the model, the data of the test set and the validation set were verified by a trained classifier, and the prediction results are output. Then we use the pROC package and plot function of the R language to display the ROC curve of the data set to evaluate the effectiveness of the model.

After the construction of the classifier and the evaluation of the classification efficiency, we found that the classifier constructed by the SVM algorithm has a slight advantage over the classifier constructed by the Decision tree algorithm (Figures 2B,C). In order to measure the generalization ability of the support vector machine model, we introduced fivefold cross-validation. We found that the AUC value of the fivefold verification result is stable (Supplementary Figure S1), indicating that the choice of hyperparameters of the model is excellent. We obtained excellent results in differentiating periodontitis from normal tissue from the perspective of immune cells, suggesting that the disruption of the immune microenvironment of the gingival tissue is an important cause of periodontitis. Further, the exploration of the molecular mechanisms underlying the formation of the immunosuppressive microenvironment in periodontitis is crucial.

### Immune System Imbalance at the Molecular Level

#### Statistical Analysis of Gene Expression Matrix

**FIGURE 3:**
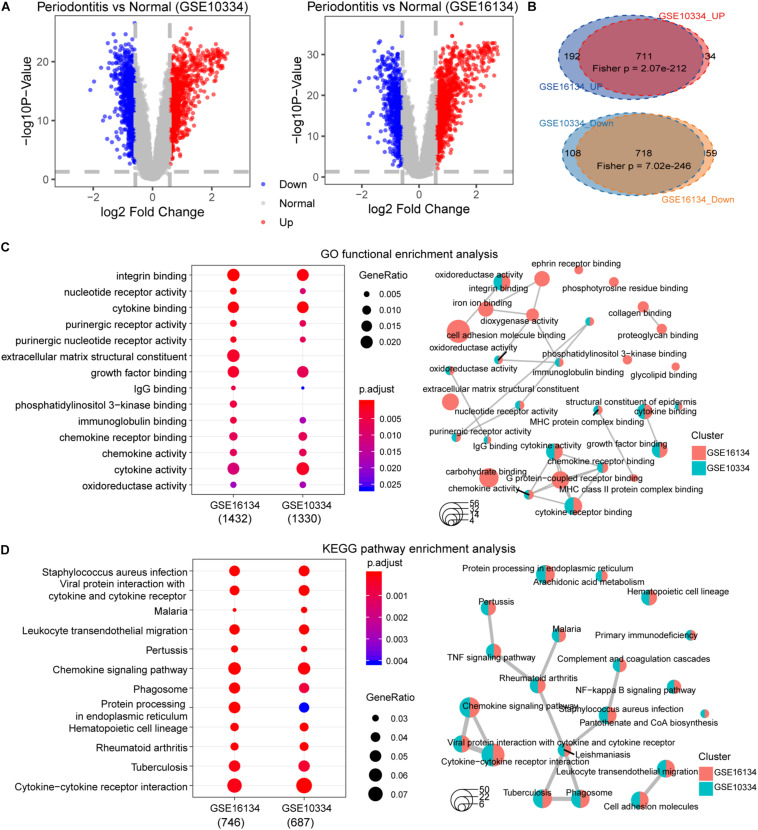
Differential expression analysis and functional enrichment analysis between periodontitis and normal samples. **(A)** This picture represents the volcano map of DEGs for the GSE10334 series. **(B)** This venn diagram describe the intersection of the up- and down-regulated genes in the GSE10334 and GSE16134 series. Fisher’s exact test is used to measure the significance level of overlap. **(C–D)** This picture represents the dotplot and emapplot of the GO function enrichment node of DEGs in the GSE10334 and GSE16134 series of samples. e represents the dotplot and emapplot of the DEGs KEGG pathway enrichment in GSE10334 and GSE16134 series samples.

First, we performed statistical tests on the expression profile data of the GSE10334 and GSE16134 series with abundant sample sizes, and calculated two test indicators *P*-value and Fold Change. We obtained 1,571 and 1,680 DEGs from the two series of GSE10334 and GSE16134, respectively (Figures 3A,B). From the results, we found that there are a large number of DEGs between periodontitis samples and normal samples. In order to evaluate the reliability of the experimental data, we tested the overlap levels of the up-regulated and down-regulated genes in GSE10334 and GSE16134, respectively. We found that the up-regulated and down-regulated genes in GSE10334 and GSE16134 have significant overlap, indicating that the DEGs we obtained from the analysis of experimental data are reliable (Figure 3C). Further, there are 1,424 DEGs shared by GSE10334 and GSE16134.

Next, we conduct preliminary statistics on the functional effects of DEGs. These two series of DEGs are integrated into a multi-gene set list, which is used for multi-gene set GO function enrichment and KEGG pathway enrichment, and the functional pathway with *p* < 0.05 is selected as the significant function. We use dotplot and emapplot to display 15 functional nodes and pathways in the results of function and pathway enrichment (Figure 3D). Since the DEGs of the two series of samples have a large overlap, they are very similar in function and pathway enrichment. We can see from the enrichment results that periodontitis disease has significant enrichment in cell growth and related immune functions. And which DEGs interact and regulate relationships deserve further analysis.

#### PPI Network of DEGs

**FIGURE 4:**
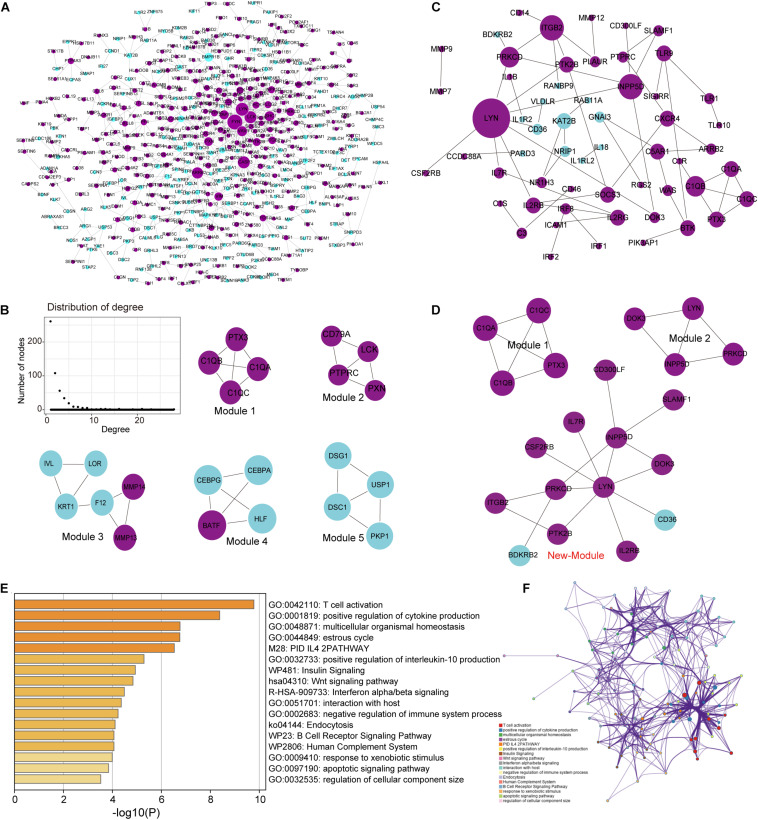
Analysis of the topological properties and functional modules of the PPI network of DEGs and crosstalk genes. **(A)** This picture represents the protein interaction network of two series of integrated DEGs. There are 647 relationship pairs and 515 nodes in the network. **(B)** This picture is a moderate topological analysis of the PPI network of DEGs and the five functional modules in the network. **(C)** This picture shows the PPI network of crosstalk gene, which has 58 relational pairs and 57 nodes. **(D)** This picture is the three modules in the PPI network of crosstalk gene. **(E)** Bar graph of enriched terms across TF and target genes associated with immune pathways, colored by p-values. **(F)** Network of enriched terms colored by cluster ID, where nodes that share the same cluster ID are typically close to each other.

Building a protein interaction network (PPI) is a common method to reveal the interaction relationships and functional modules between genes, so we constructed a PPI network of DEGs (Figure 4A). First, merge these two series of DEGS to obtain a total of 1,822 DEGs, and then extract the corresponding interaction relationship pairs to draw the PPI network. In the biological network, the node with the higher degree plays a bigger role in the network and has important functions. Therefore, we extracted the top 30 degree-ranked genes as important periodontitis disease-related genes (Supplementary Table S2). The results show that genes such as FYN, LYN, LCK, Critical Assessment of Techniques for Protein Structure Prediction experiment (CASP3), arrestin beta 2 (ARRB2) are the central node genes with high connectivity in the PPI network. Among them, FYN, LYN, and LCK are all members of the protein tyrosine kinase (PTK) family, and they are non-receptor PTKs. Studies have shown that most proto-oncogenes have PTK activity, and their abnormal expression will lead to disorders of cell proliferation and eventually tumorigenesis (Drake et al., 2014). Non-receptor PTK-mediated signal transmission plays an important role in the activation of T cells, B cells, NK cells and granulocytes, and the abnormality of its gene structure or gene expression is the cause of certain immunodeficiency diseases and immunoproliferative diseases (Vivier et al., 2004; Vasquez et al., 2019). This means that FYN, LYN and LCK, which are highly expressed, play an important role in the imbalance of the immune system of periodontitis. In addition, we selected five important functional modules from the PPI network (Figure 4B), all of which play an important role in cellular immunity (Module 1) and cell growth and proliferation. In order to further study the relationship between immunity and periodontitis disease, we extracted genes related to immunity among DEGs and conducted a series of analysis and research.

#### Crosstalk Gene in Immune Imbalance

Since crosstalk occurs when TFs regulate multitude of immune-related genes in periodontitis disease, it is intriguing to explore the regulatory mechanisms of immune-related genes (Friedlander et al., 2016; Grah and Friedlander, 2020). We extracted the immune-related genes from the DEGs and defined them as crosstalk genes. Then, we obtained 159 crosstalk genes, which are immune-related genes differentially expressed in periodontitis diseases. We extracted the PPI relationship pairs of these crosstalk genes to draw a PPI interaction network, and analyzed the functional modules and topological properties of the network (Figure 4C). We obtained 3 functional modules including a new immune function module (New-module) which was not recognized in the previous PPI network (Figure 4D).

As we all know, TFs can control gene expression and expression efficiency (Lambert et al., 2018). Therefore, the analysis of transcription regulation relationship helps us understand the process of several gene expression changes. We collected TF-target relationships from TRRUST and ORTI database which identify TF-target regulations from small-scale experimental studies and interrogating gene expression data. These TF-target relationships were mapped to the transcriptional regulatory network of DEGs associating with crosstalk genes (Supplementary Figure S2). There were 19 TFs in this transcriptional regulatory network, of which 14 were up-regulated and 5 were down-regulated. A total of 5 TFs were crosstalk genes that had unbinding event with known target genes, and they were all up-regulated in expression, including early growth response 1 (EGR1), ETS proto-oncogene 1 (ETS1), interferon regulatory factor 4 (IRF4), RUNX family transcription factor 3 (RUNX3), and X-box binding protein 1 (XBP1). We combined immune-related genes on the basis of transcriptional regulatory network to explore the functions of TFs in the immune microenvironment according to Metascape (Zhou et al., 2019). We found that these TFs and their targeted genes are closely related to the activity of T cells (Figures 4E,F), which may lead to the formation of periodontitis immunosuppressive microenvironment. By analyzing the topological properties of the network (Table 2 and Supplementary Table S3), we found that EGR1, ETS1, RUNX3, and XBP1 were associating with multiple genes. We also found that most of the up-regulated genes in the New-module functional module of the cross-talk gene PPI network are regulated by ETS1 and EGR1.

**TABLE 2:** Top 10 outdegree genes in the transcriptional regulatory network as key genes.

Symbol	Out degree	Average shortest path Length	Betweenness centrality	Closeness centrality	Regulatory_type	EXP_type
ETS1	859	1.022	0.001	0.979	TF_Target	Down
EGR1	41	1	3.23E-05	1	TF_Target	Down
RUNX3	8	1	1.55E-05	1	TF_Target	Down
XBP1	6	1	1.29E-06	1	TF_Target	Down
CEBPA	5	1	0	1	TF	Down
IRF1	2	1	8.60E-07	1	TF_Target	Up
IRF2	2	1	8.60E-07	1	TF_Target	Up
POU2F2	2	2.018	1.29E-06	0.495	TF_Target	Down
STAT4	2	1.333	0	0.750	TF_Target	Down
IRF4	1	1	3.87E-06	1	TF_Target	Up

#### Explore the Immune Function of New-Module

**FIGURE 5:**
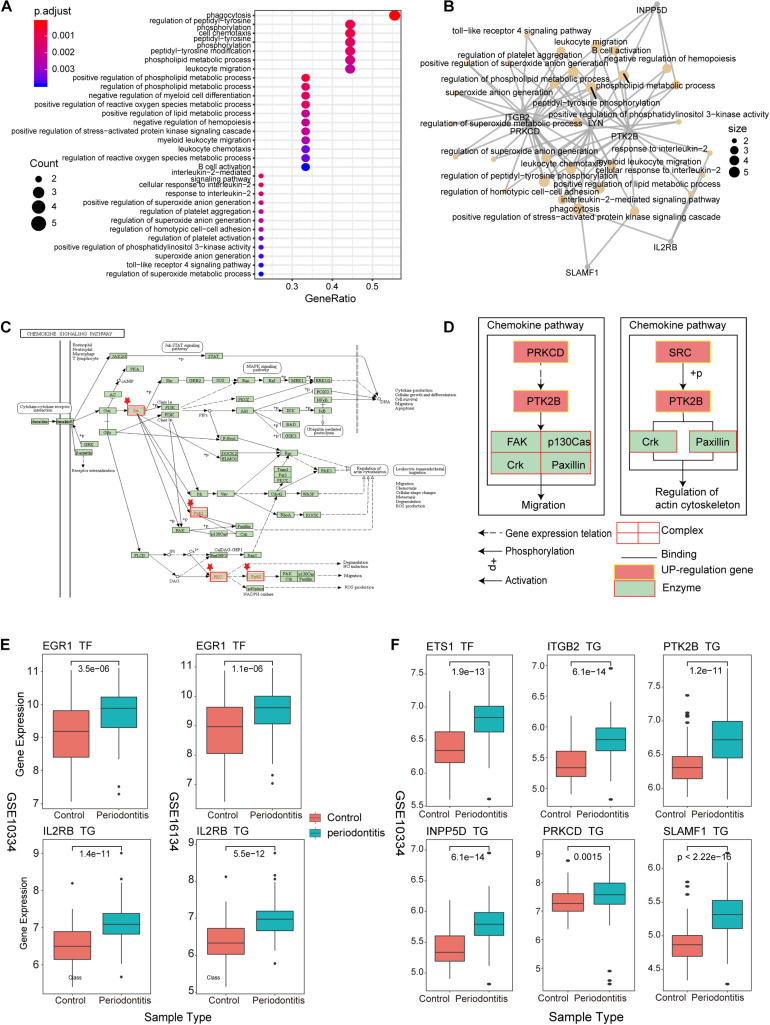
New-module function and pathway analysis **(A)**. The dotplot of enriched biological pathways (BP) across up-regulated genes in the New-module. GeneRatio is the number of enriched genes/number of all genes of a GO term. **(B)** Network of enriched terms, where nodes that share the same genes are typically link to each other. The size of the dot represents the counts of gene. **(C)** The pathway diagram is one of the functional pathways enriched by up-regulated genes in the New-module gene. **(D)** The mechanism of the New-module up-regulated genes on the chemokine signaling pathway. **(E)** The boxplot represents the transcriptional regulatory relationship of the up-regulated genes in the New-module for GSE10334 and GSE16134 series. **(F)** The same as in **(E)** but only for GSE10334 series.

As an important and novel functional module, New-module is worthy of our in-depth exploration. We extracted the up-regulated genes in New-module as a gene set, and analyzed their biological pathways (BP) and functional pathways, where ont = ‘BP’ was set in enrichGO, and *p* < 0.05 was set uniformly. Through enrichment analysis of the up-regulated target genes in module3, we have obtained significantly enriched functional pathways. For the large number of BPs, we used dotplot and cnetplot to show only the top 30 BPs terms (Figures 5A,B). These BPs are mainly related to immune cell invasion and activity. In the cnetplot, we found that these biological pathways mainly involve 7 genes, including INPP5D, LYN, PRKCD, PTK2B, ITGB2, SLAMF1, and IL2RB. These genes are only significantly enriched in one pathway, namely the Chemokine Signaling pathway (hsa04062; chemokine signaling pathway), in which three genes including LYN, PRKCD and PTK2B are involved (Figures 5C,D). Studies have found that chemokines play a basic role in the transport and activation of monocytes and lymphocytes in the inflammation site. For example, this mechanism can perpetuate local inflammation in the joints of RA patients (Zhang et al., 2015). So, in periodontitis disease, it was possible to believe that the production and persistence of inflammation caused by immunosuppressive microenvironment is achieved through the influence on chemokine signaling pathways.

We then used boxplot to show the relationship between these genes and the expression of TFs, and we found that the expression changes of TF and target genes are consistent, which is in line with the transcription regulation relationship (Figures 5E,F). The TFs involved are the two high-outdegree TFs, ETS1 and EGR1, which reveals that the TFs ETS1 and EGR1 play a crucial role in the invasion and activity of immune cells in periodontitis. Further, we explored whether these TFs played driver roles in TF-target relationships by using Chromatin Immunoprecipitation Sequencing (ChIP-seq) data from ENCODE (v112). Enriched sequencing read peaks of these TFs have been found in the transcription factor binding site (TFBS) regions of downstream target genes. For example, the EGR1-IL2RB relationship of Figure 5E has been supported by multiple ChIP-seq datasets (Supplementary Figure S3). The ETS1-target relationships of Figure 5F has also been supported by multiple ChIP-seq datasets (Supplementary Figures S4–S8). Since the immune function module New-module plays an important role in periodontitis disease, we decided to rebuild the classifier using the gene of this module as features and compare the performance of the previous classifiers.

### Construct a Classifier Based on New-Module

**FIGURE 6:**
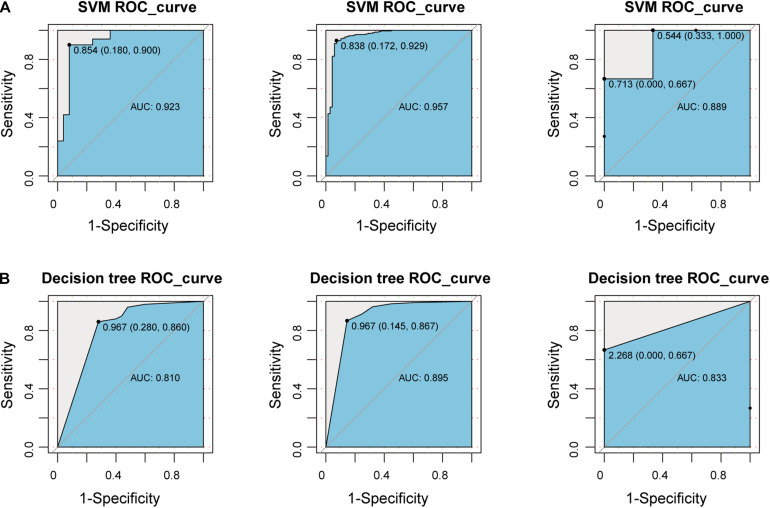
Construct a classifier based on the New-module gene. **(A)** The ROC curves of the test set and validation set for SVM algorithm constructed with the New-module functional module gene in the crosstalk gene PPI network. **(B)** The same as in **(A)** but for the decision tree algorithm.

To explore whether the new-module can accurately define periodontitis and normal tissue, we constructed classifiers using the genes in the module as features. Considering that the expression values of the GSE10334, GSE16134, and GSE23586 series are of different magnitudes, we normalized them to make them consistent. We built two classifiers based on decision tree and SVM and used the classifier to predict the test set and the validation set (see section “Materials and Methods”). We found that the classifier constructed with SVM is the best here, and the AUC values of the test set and the two validation sets are 0.923, 0.957, and 0.889, respectively (Figure 6). The lower AUC value of GSE23586 as the test set is caused by the small sample size. Generally speaking, the effect of the classifier is better.

Then we compared the performance evaluation results of this classifier with the previous ones (Table 3). From the comparison results, we can clearly see that the effect of constructing a classifier based on the new-module functional module is better than based on the different content of immune cells. All these suggesting that although there are differences in the fraction of immune cells between periodontitis samples and normal samples, the differences will be more significant at the level of molecular level.

**TABLE 3:** Comparison table of performance evaluation of two classifiers successively.

Classification features	Series number	data sets	SVM AUC	Decision tree AUC
Immune cells	GSE10334	Test set	0.815	0.656
	GSE16134	Validation set	0.918	0.855
	GSE23586	Validation set	0.889	0.833
Important crosstalk genes	GSE10334	Test set	0.923	0.810
	GSE16134	Validation set	0.957	0.895
	GSE23586	Validation set	0.889	0.833

## Discussion

In this study, we systematically analyzed the immune imbalance of periodontitis from the cellular to molecular level. Measuring the fraction of immune cells between periodontitis and normal tissues was used to determine the feature and role of immune cells in periodontitis. Statistical analysis of gene expression profiles is used to reveal abnormally expressed genes in periodontitis. The PPI was constructed to explore potential functional modules and reveal new molecular mechanisms of immune imbalance in periodontitis. We have reconstructed the PPI network base on immune genes and discovered a new immune function module named New-module. By integrating TF-target relationships and ChIP-seq data, we found that EGR1, ETS1, RUNX3, and XBP1 were key TFs that regulate the expression of genes that participate in the formation of the immunosuppressive microenvironment. The up-regulated genes are mainly regulated by EGR1 and ETS1 in New-module. In addition, New-module not only plays an important role in the imbalance of the immune system, but is also closely related to the occurrence and persistence of periodontal tissue inflammation.

Periodontitis is mainly a chronic inflammation of periodontal tissue caused by pathogens, which has the characteristics of complicated pathogenesis and long duration. Previous studies have shown that the imbalance of the immune system caused by pathogen colonization is an important factor in the occurrence and development of periodontitis. The majority of work has focused on the external pathogenic factors and clinical treatment of periodontitis, with limited documentation of indications that the changes in the molecular mechanism of the immune system of patients with periodontitis. In addition, more and more studies have demonstrated the significance of the imbalance of the immune system for periodontitis, including the abnormality of cytokines in the host immune response (Pan et al., 2019), and the immune imbalance of periodontitis leads to systemic inflammation (Hajishengallis, 2015). Exploring the disease tissue microenvironment at single-cell resolution is a popular direction, but the lack of high-throughput data for periodontitis has forced us to consider other approaches. In order to be able to further explore the tissue microenvironment and epigenetic characteristics of periodontitis in future research, TOAST (Li et al., 2019, 2020; Li and Wu, 2019) tool that offers functions for detecting cell-type specific differential expression (csDE) and differential methylation (csDM) brings convenience to our research. In the current study, we comprehensively assessed the immune system imbalance of periodontitis from the cellular to molecular level, which gained a new insight in protein interaction and transcriptional regulation.

During the construction of PPI networks, usage of immune genes only will lose many other pathway signals. Our purpose is to explore the molecular mechanism of the immune microenvironment reprogramming of periodontitis disease. Although our selection of immune genes will ignore other signaling pathways, the formation mechanism of the immunosuppressive microenvironment of periodontitis disease is important. In the future, we will integrate more genes into the PPI networks and perform functional analysis to characterize periodontitis disease comprehensively.

We successfully determined the immunosuppressive microenvironment of periodontitis in the measurement of immune cell distribution. Notably, we measured the distribution of immune cells and differential gene expression in two series with rich samples, which can effectively avoid the false negative problem faced in the research. Our data may discover previously overlooked pathogenic genes and molecular mechanisms, adding a new blueprint for periodontitis research. In addition, we also used a machine learning algorithm to build a classifier model to consider the reliability and pros and cons of the statistically obtained disease characteristics. Periodontitis is mainly a local inflammation caused by pathogen-induced immune invasion. Therefore, investigation and interpretation of the immune system would provide novel and useful insights into the mechanisms underlying the functions of these molecules in periodontitis. In our further work, we will perform experiments *in vitro* to validate key regulators identified from our results. The experimental strategy will measure the expression levels of risk genes using qRT-PCR in normal and disease tissues. Further, siRNAs will be used to knockdown their expression and study gene functions with cell proliferation assay, wound healing assay.

## Conclusion

In summary, we provide a comprehensive view of the imbalance mechanism of the periodontitis immune system from the cellular to the molecular level. Our findings expand existing knowledge about immunosuppressive associated with periodontitis. The integration of multi-platform data comprehensively reveal that the immune system imbalance mechanism of periodontitis patients enhances the interpretability of the pathogenesis of periodontitis, which may help the development of new periodontitis treatments.

## Data Availability Statement

The original contributions presented in the study are included in the article/Supplementary Material, further inquiries can be directed to the corresponding author/s.

## Author Contributions

LB and LH conceived and designed the experiments. LL, TL, and DZ analyzed the data. JD and BW collected the data. LH and LL validated the method and data. LH wrote this manuscript. All authors read and approved the final manuscript.

## Conflict of Interest

The authors declare that the research was conducted in the absence of any commercial or financial relationships that could be construed as a potential conflict of interest.

## Footnotes

1 https://www.ncbi.nlm.nih.gov/geo/

2 http://www.disgenet.org

3 http://biokb.nb.org/HisgAtlas/

4 https://www.drugbank.ca/

5 http://thebiogrid.org/

6 http://mint.bio.uniroma2.it/mint/

7 http://www.hprd.org/

8 https://www.ebi.ac.uk/intact/

9 http://ophid.utoronto.ca/ophidv2.204/

10 https://www.innatedb.ca/

11 https://www.grnpedia.org/trrust/

12 http://orti.sydney.edu.au/about.html
