# Supplementary material for: Exploring the Imbalance of Periodontitis Immune System From the Cellular to Molecular Level
Source: Front Genet. 2021 Mar 26;12:653209. doi: 10.3389/fgene.2021.653209 (PMC8033214; doi:10.3389/fgene.2021.653209)
Supplement: Supplementary file 2 [file Data_Sheet_1.docx]

Supplementary Figures


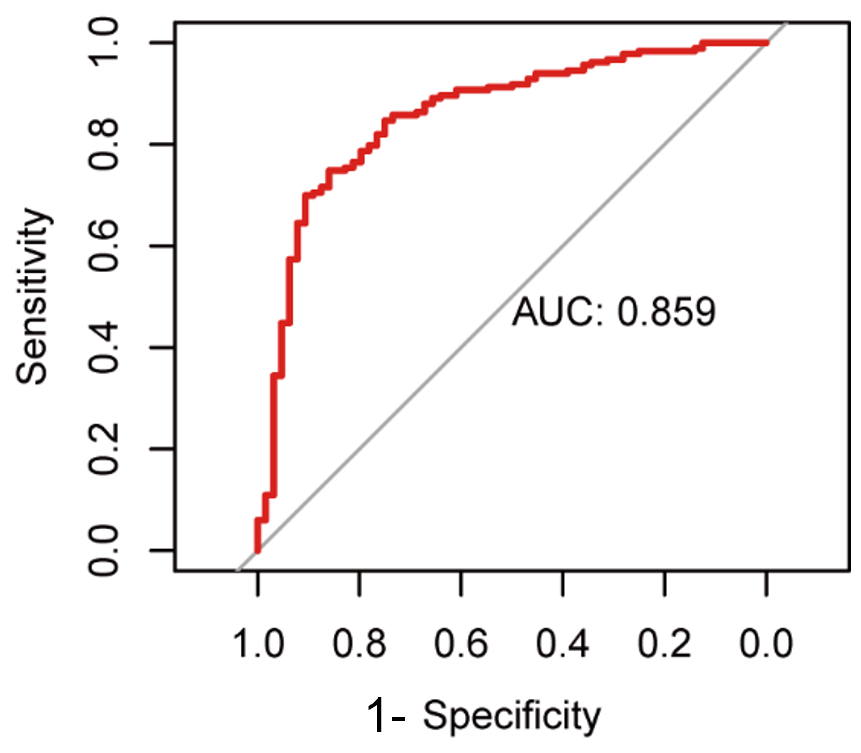


**Figure S1.** We introduced 5-fold cross-validation to evaluate the generalization ability of the model. We integrated the results of the 5-fold inspection to draw the ROC curve and indicated the AUC value.

*
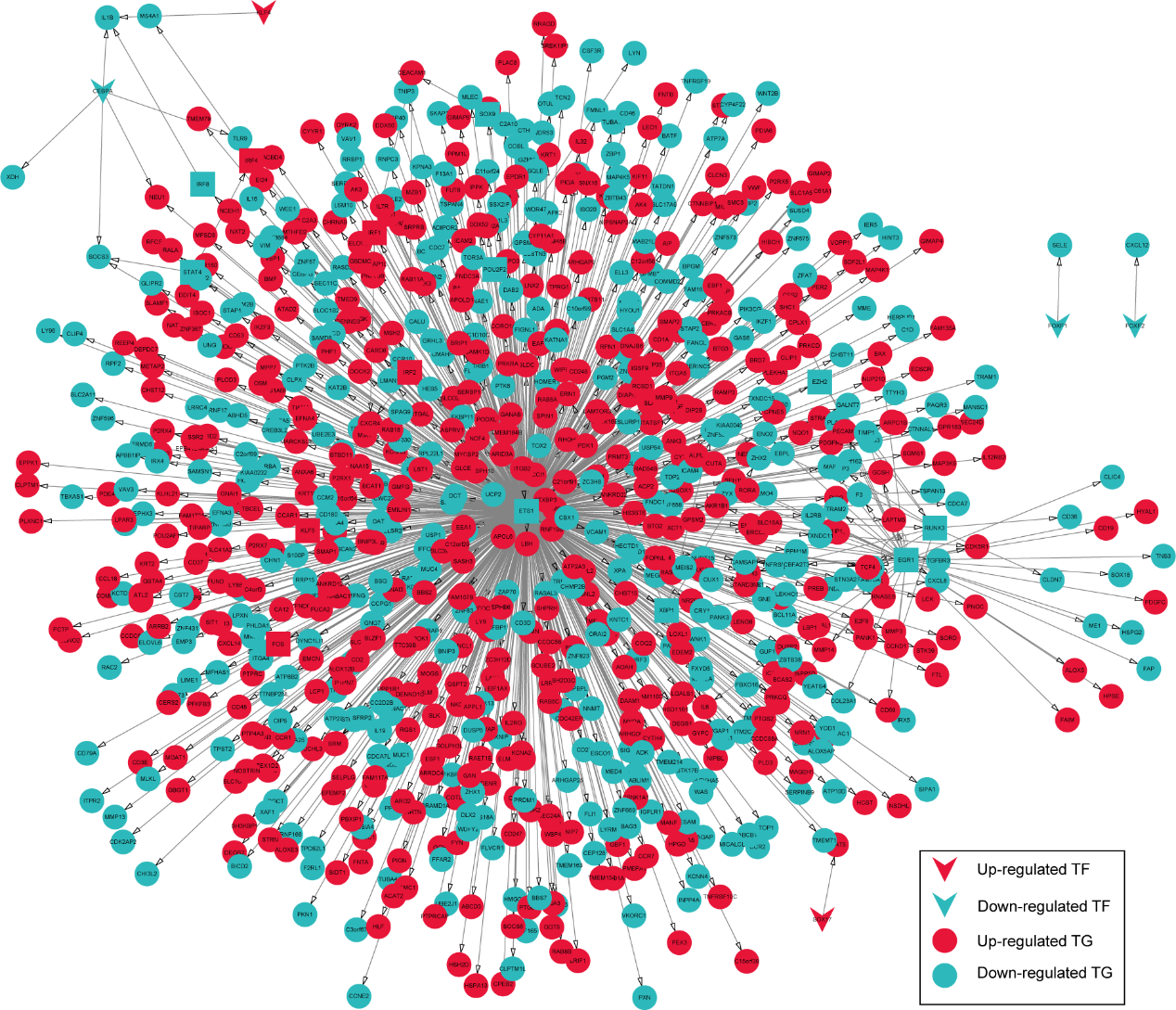
*

**Figure S2.** This figure shows the transcriptional regulation of crosstalk genes. The red represents up-regulated genes, blue represents down-regulated genes, circles represent target genes, arrow shapes represent transcription factors, and rectangles represent both target genes and transcription factors.


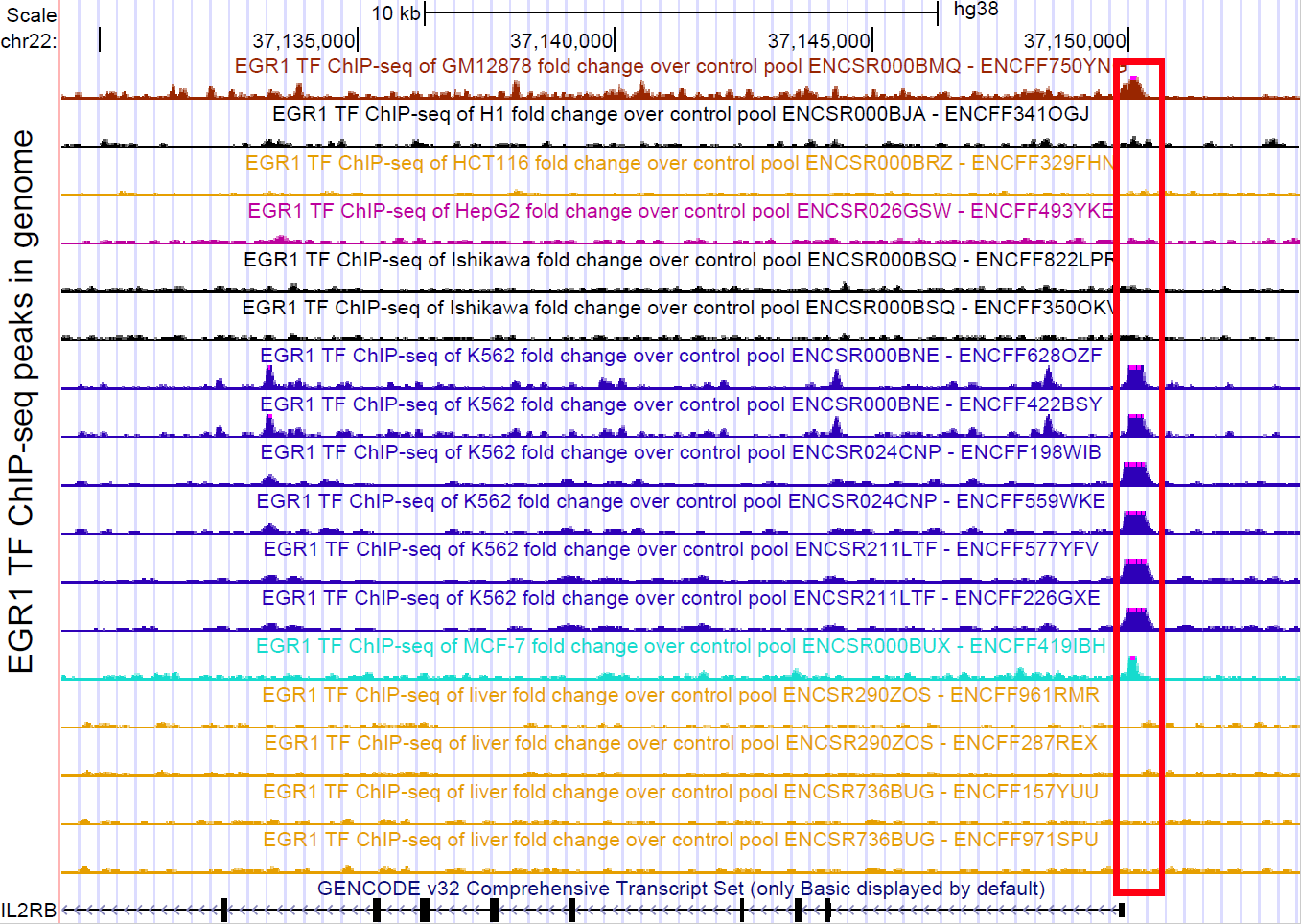


**Figure S3.** Enriched sequencing read peaks of EGR1 in the TFBS region of IL2RB based on multiple ChIP-seq datasets.


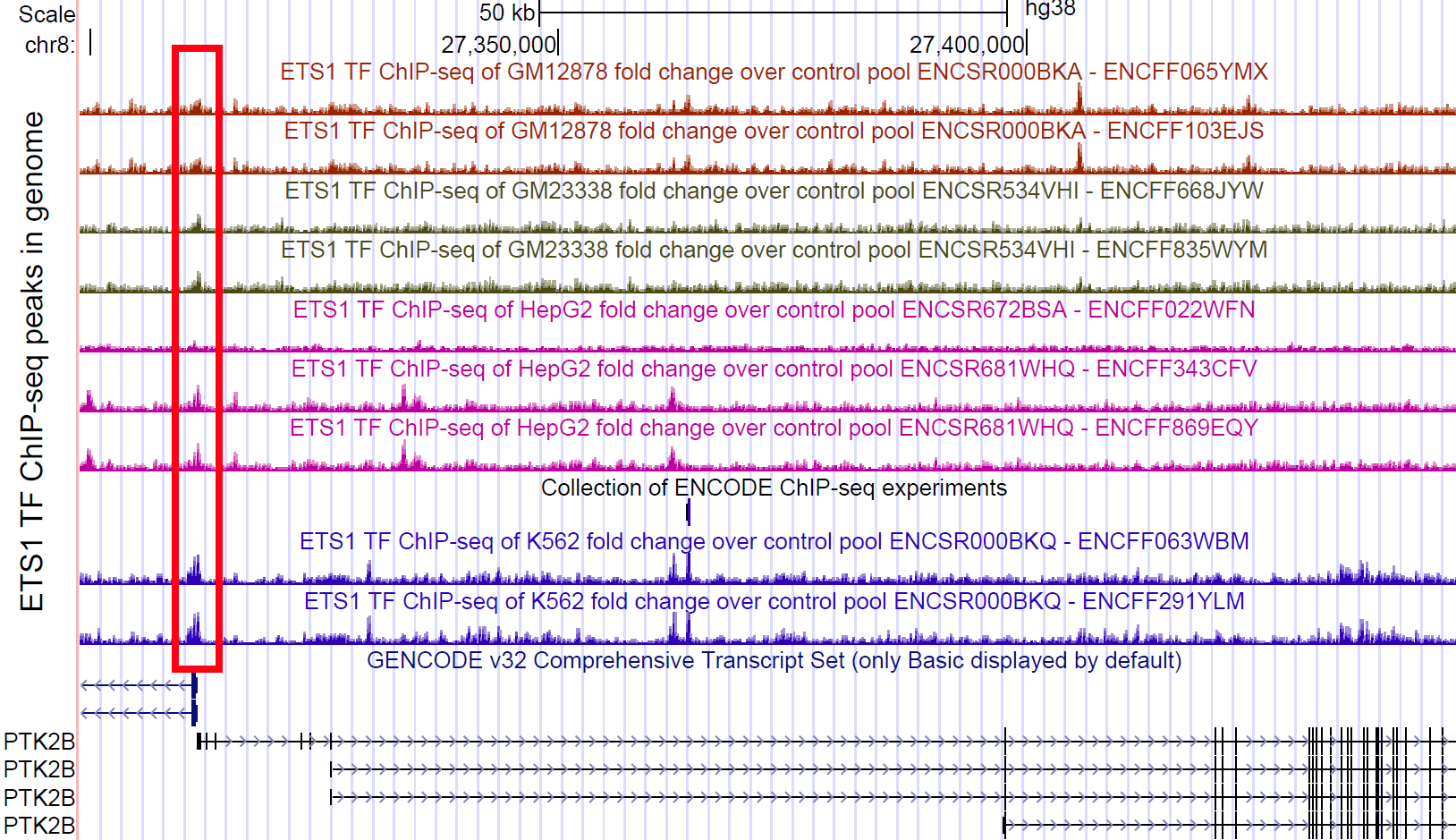


**Figure S4.** Enriched sequencing read peaks of ETS1 in the TFBS region of PTK2B based on multiple ChIP-seq datasets.


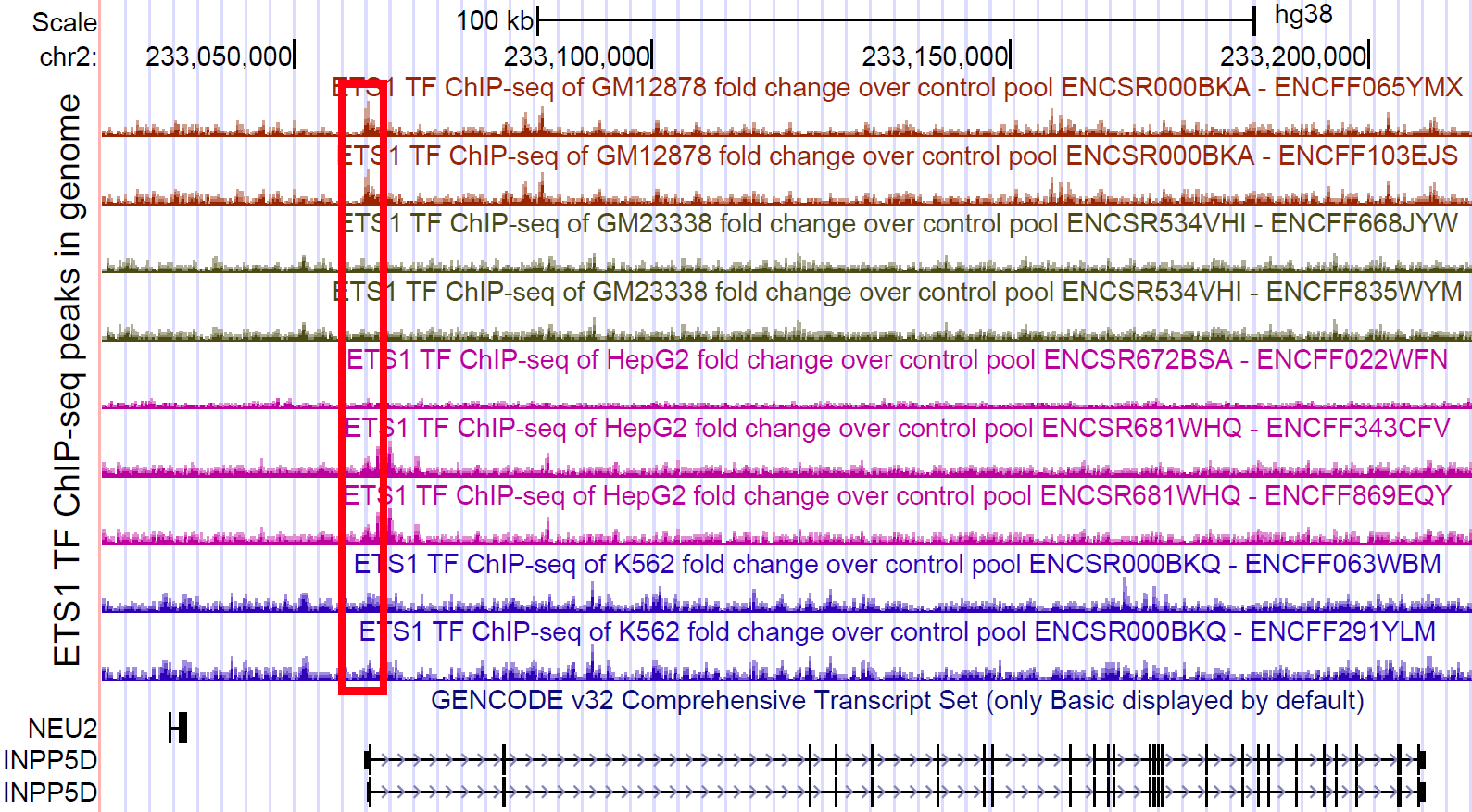


**Figure S5.** Enriched sequencing read peaks of ETS1 in the TFBS region of INPP5D based on multiple ChIP-seq datasets.


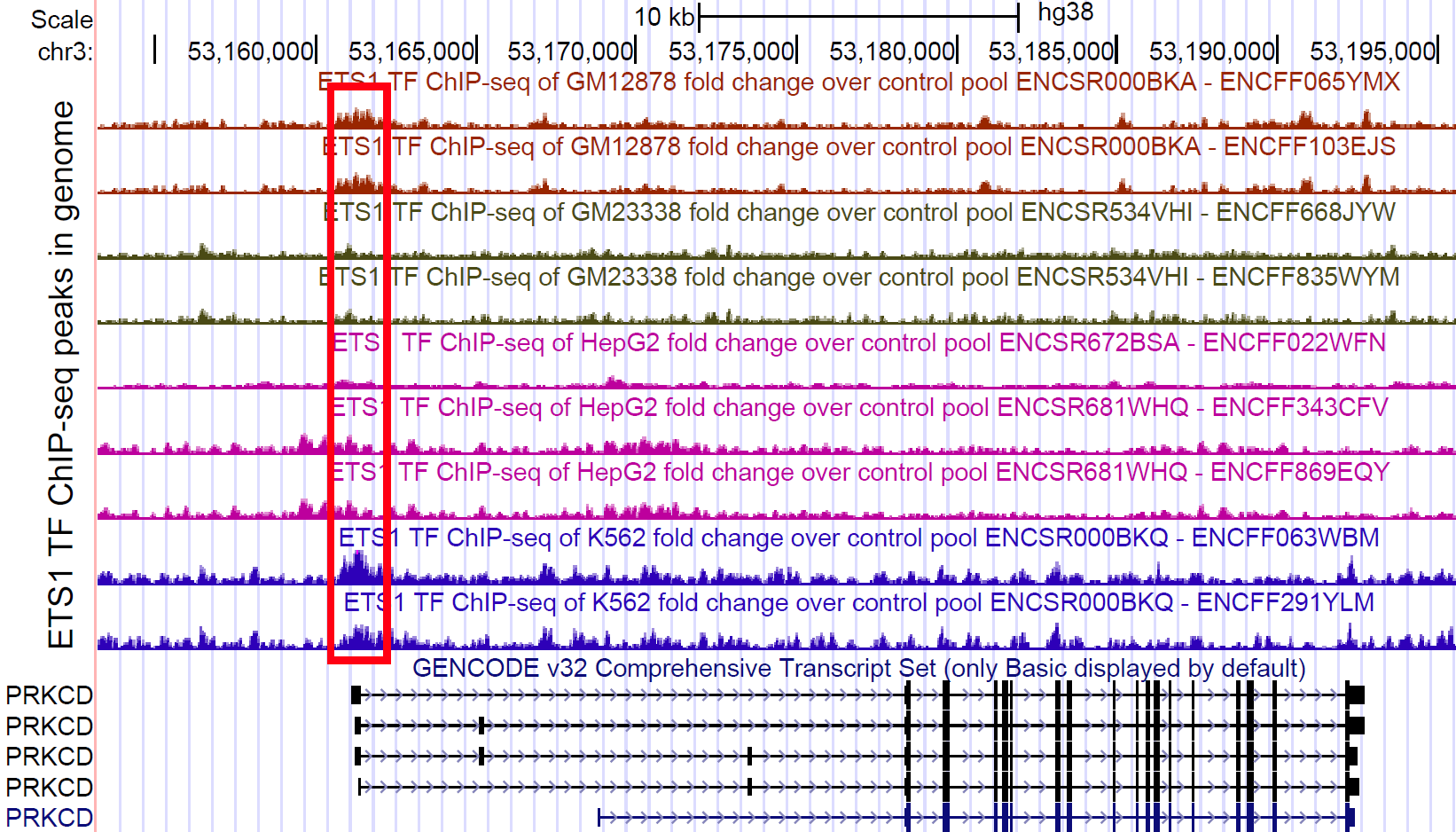


**Figure S6.** Enriched sequencing read peaks of ETS1 in the TFBS region of PRKCD based on multiple ChIP-seq datasets.


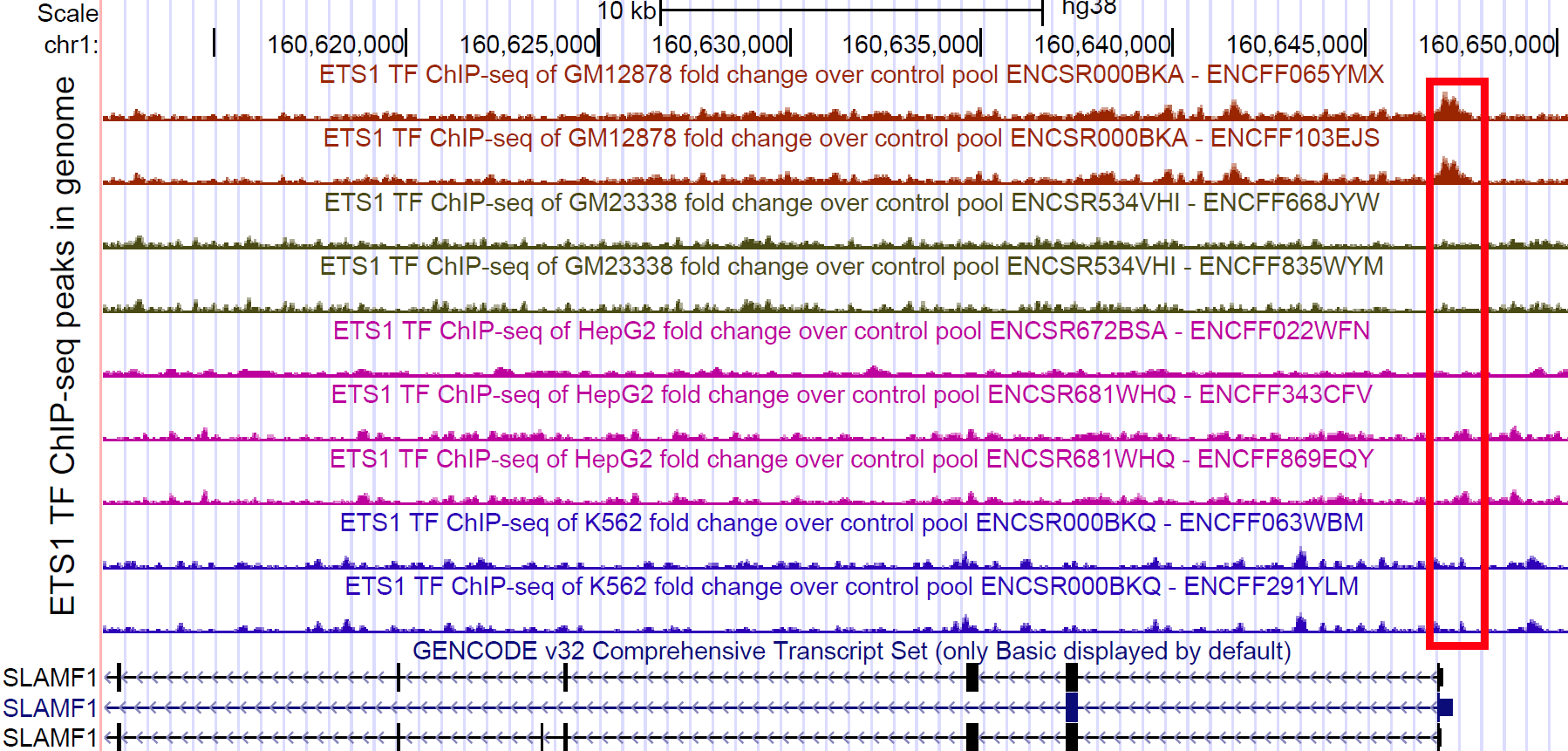


**Figure S7.** Enriched sequencing read peaks of ETS1 in the TFBS region of SLAMF1 based on multiple ChIP-seq datasets.


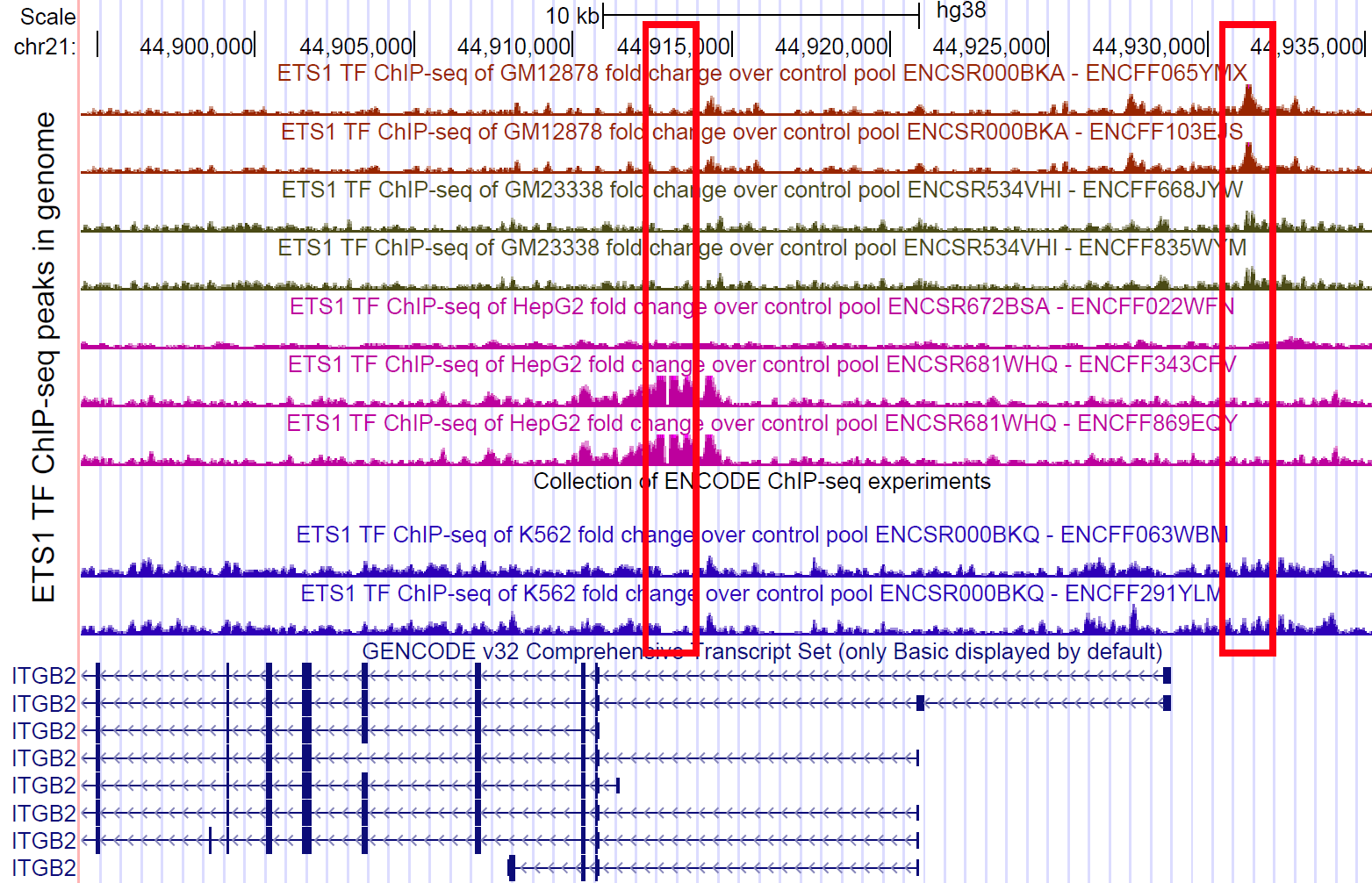


**Figure S8.** Enriched sequencing read peaks of ETS1 in the TFBS region of ITGB2 based on multiple ChIP-seq datasets.

Supplementary Tables

**Table S2: The top 30 degree-ranked genes in the DEGs PPI network**

| SYMBOL | Drgee | AverageShortestPathLength | BetweennessCentrality | ClosenessCentrality | ClusteringCoefficient | TopologicalCoefficient |
| --- | --- | --- | --- | --- | --- | --- |
| FYN | 28 | 3.628 | 0.155 | 0.276 | 0.063 | 0.066 |
| LYN | 23 | 3.938 | 0.122 | 0.254 | 0.024 | 0.069 |
| LCK | 21 | 3.968 | 0.078 | 0.252 | 0.081 | 0.088 |
| CASP3 | 21 | 3.605 | 0.228 | 0.277 | 0.029 | 0.065 |
| ARRB2 | 19 | 3.913 | 0.191 | 0.256 | 0.000 | 0.066 |
| SHC1 | 16 | 4.143 | 0.086 | 0.241 | 0.042 | 0.095 |
| VAV1 | 16 | 3.670 | 0.162 | 0.272 | 0.058 | 0.089 |
| ZAP70 | 14 | 4.175 | 0.037 | 0.240 | 0.088 | 0.110 |
| PXN | 13 | 3.688 | 0.172 | 0.271 | 0.077 | 0.104 |
| BMPR1B | 13 | 5.288 | 0.070 | 0.189 | 0.000 | 0.077 |
| PRKCD | 12 | 4.325 | 0.056 | 0.231 | 0.045 | 0.121 |
| VIM | 12 | 3.960 | 0.199 | 0.253 | 0.030 | 0.097 |
| PTK2B | 11 | 4.188 | 0.038 | 0.239 | 0.036 | 0.120 |
| ITGB2 | 10 | 4.295 | 0.037 | 0.233 | 0.044 | 0.123 |
| HCK | 9 | 4.403 | 0.014 | 0.227 | 0.056 | 0.170 |
| PLCG2 | 9 | 4.300 | 0.009 | 0.233 | 0.167 | 0.185 |
| BTK | 9 | 4.230 | 0.061 | 0.236 | 0.083 | 0.151 |
| CD247 | 9 | 4.345 | 0.025 | 0.230 | 0.167 | 0.157 |
| INPP5D | 9 | 4.570 | 0.027 | 0.219 | 0.111 | 0.150 |
| MME | 8 | 4.908 | 0.076 | 0.204 | 0.000 | 0.140 |
| FOS | 8 | 5.383 | 0.051 | 0.186 | 0.000 | 0.135 |
| PTPRC | 8 | 3.990 | 0.028 | 0.251 | 0.250 | 0.186 |
| GNAI3 | 8 | 4.943 | 0.020 | 0.202 | 0.000 | 0.188 |
| MMP9 | 8 | 4.990 | 0.075 | 0.200 | 0.000 | 0.163 |
| MAPK13 | 7 | 5.573 | 0.021 | 0.179 | 0.000 | 0.200 |
| TUBA4A | 7 | 3.980 | 0.050 | 0.251 | 0.095 | 0.187 |
| VCAN | 7 | 4.958 | 0.044 | 0.202 | 0.095 | 0.183 |
| PF4 | 7 | 5.295 | 0.040 | 0.189 | 0.095 | 0.188 |
| CD79A | 7 | 4.230 | 0.024 | 0.236 | 0.238 | 0.211 |
| GNAI1 | 7 | 4.858 | 0.019 | 0.206 | 0.000 | 0.204 |

**Table S3: Top 10 indegree genes in the transcriptional regulatory network**

| SYMBOL | Indegree | AverageShortestPathLength | BetweennessCentrality | ClosenessCentrality | Regulatory_type | EXP_type |
| --- | --- | --- | --- | --- | --- | --- |
| CXCR4 | 4 | 0 | 0 | 0 | Target | Up |
| CCND1 | 3 | 0 | 0 | 0 | Target | Up |
| IL6 | 3 | 0 | 0 | 0 | Target | Up |
| TCF4 | 3 | 0 | 0 | 0 | Target | Up |
| TIMP1 | 3 | 0 | 0 | 0 | Target | Down |
| CEACAM1 | 3 | 0 | 0 | 0 | Target | Up |
| IL1B | 3 | 0 | 0 | 0 | Target | Down |
| SOCS3 | 3 | 0 | 0 | 0 | Target | Down |
| EGR1 | 2 | 1 | 3.23E-05 | 1 | TF_Target | Down |
| AKR1B1 | 2 | 0 | 0 | 0 | Target | Up |
